# Simvastatin Effect on Calcium and Silicon Plasma Levels in Postmenopausal Women with Osteoarthritis

**DOI:** 10.1007/s12011-016-0635-1

**Published:** 2016-02-09

**Authors:** Anna Horecka, Anna Hordyjewska, Tomasz Blicharski, Joanna Kocot, Renata Żelazowska, Anna Lewandowska, Jacek Kurzepa

**Affiliations:** Chair and Department of Medical Chemistry, Medical University of Lublin, Chodźki 4A, PL 20-093 Lublin, Poland; Chair of Orthopedics and Rehabilitation, Medical University of Lublin, Jaczewskiego 8, PL 20-954 Lublin, Poland

**Keywords:** Osteoarthritis, Postmenopausal women, Simvastatin, Plasma bioelements, HPLC

## Abstract

Postmenopausal women more often suffered from knee osteoarthritis and its pathogenesis still remains unclear. Calcium and silicon are significant elements involved in bone and joint metabolism, especially in older people. Cardiovascular diseases are common worldwide and simvastatin is the most prescribed drug in such population of patients. The purpose of this study was to evaluate the effect of simvastatin administration on calcium and silicon concentration in the plasma of postmenopausal women with osteoarthritis. Sixty postmenopausal mild hypercholesterolemic women (mean age 61.4 years, range 54–68) were enrolled. Thirty patients received simvastatin (20 or 40 mg/day) for at least 1 year before being enrolled (simvastatin “+” group). Control group consists of remaining 30 women (simvastatin “−“group). Silicon and calcium concentrations were measured spectrophotometrically. Plasma simvastatin level was determined 3 h after the drug administration using HPLC-UV-Vis. Calcium but not silicon level was significantly lower in patients receiving simvastatin in comparison with non-statin group (1.91 ± 0.32 vs. 2.33 ± 0.19 mmol/l, *p* < 0.05). A weak but significant positive correlation between plasma silicon and simvastatin levels (*r* = 0.3, *p* < 0.05) was observed; this may be due to the fact that simvastatin contains silicon dioxide as an inactive ingredient. The mean simvastatin concentration was 9.02 ng/ml. All hypotheses were verified at the significance level of *p* < 0.05. A statistically significant decrease in the plasma calcium concentration of postmenopausal women, treated with simvastatin suggests that simvastatin may play a role in calcium metabolism in postmenopausal women with osteoarthritis. Positive correlation of simvastatin concentration with silicon level in the plasma suggests that both might prompt the positive effect of osteoarthritis treatment.

## Introduction

Osteoarthritis is the most common joint disease that occurs mainly in older people, whose physical capability is poorer [1]. Eighty percent of individuals older than 75 years suffer from osteoarthritis and its pathogenesis still remain unclear [2]. It is characterized by destruction of cartilage matrix and on the joint surface. The process of destruction is followed by osteophytes production at the joint margins, cystic degeneration, and sclerosis on the subchondral bone [3, 4]. At the synovial area, macrophages and chondrocytes release mediators that cause cartilage and bone damages [5]. In early osteoarthritis, increased production of matrix-degrading enzymes such as metalloproteinases has been noticed [4].

Statins, 3-hydroxy-3-methylglutaryl-coenzyme A (HMG-CoA) reductase inhibitors, including simvastatin, are the one of the most often prescribed drugs worldwide. The inhibition of HMG-CoA reductase by statins decreases the synthesis of mevalonate and isoprenoid units, the intermediates in pathway of cholesterol biosynthesis, leading to reduction of cholesterol production. The decrease of isoprenoid units’ synthesis is responsible also for pleiotropic activity of all statins [6]. Previous studies emphasized the relationship between statins’ activity and bone metabolism. Statins enhance the expression of the bone morphogenetic proteins (BMPs), in particular of BMP2, which results in osteoblast differentiation and finally bone formation. At the same time, statins inhibit osteoclastic activity due to decreasing of isoprenoid unit’s synthesis that is essential for osteoclastic activity [7]. In vitro and in vivo studies have demonstrated an anabolic effect of simvastatin on the bone [8]. Besides, statins have significant anti-inflammatory and immune-modulating effects. It has been reported that lipophilic statins including simvastatin, inhibit expression of interleukin-1 (IL-1) and matrix metalloproteinases (MMP-1, MMP-13) in human cartilage [5].

Adult human possesses about 1 kg of calcium in the body, mostly inside the skeleton. The involvement of calcium into the mineralization of bones is well understood [9]. The impaired calcium intake results in osteoporosis [10]. Intracellular calcium ions are responsible for signal transduction pathways including cell proliferation and cell death [11]. On the other hand, the exact role of silicon is still not clear. There is strong evidence that dietary silicon is beneficial to bone and connective tissue health [12].

The interaction between statin and calcium or silicon plasma level in not clear. Previous studies revealed the influence of rosuvastatin, but not simvastatin, on the elevation of 25-hydroxyvitamin D level resulting in the increase of calcium uptake from the digestive tract [13]. However, there is no information about the role of statins in silicon metabolism.

Menopause decreases intestinal calcium absorption [14]. It has also been suggested that silicon absorption may decrease with aging [15], although more recent work did not prove this to be the case [16]. Imbalance between bone formation and bone resorption is observed during estrogen deficiency. The increase of calcium and silicon bioavailability is an important factor in osteoporosis and osteoarthritis prevention [14].

The aim of our study was to evaluate whether chronic simvastatin treatment (20 or 40 mg/day) could affect calcium and silicon concentration in the plasma of postmenopausal women with osteoarthritis.

## Materials and Methods

### Patients

The study was approved by the Bioethical Committee of the Medical University in Lublin (No. KE-0254/294/2012) in accordance with the ethical standards laid down in the 1964 Declaration of Helsinki and its later amendments. All patients were hospitalized in the Chair of Orthopedics and Rehabilitation of the Medical University of Lublin, and they fully agreed to participate in the research constituting the present work.

Sixty postmenopausal Caucasian women with knee osteoarthritis, evolving more than 6 months, defined according to American College of Rheumatology (ACR) [17] with an average age of 61.4 (range 54–68) years were enrolled into the study. Basic classification criterion was primary slightly elevated total cholesterol level (190–240 mg/dl). The protocol of the study assumed the enrollment of two equal subgroups of patients differing with the administration of statins. Because not of all patients with slightly elevated cholesterol level in the plasma are treated with lipid-lowering drugs, we chose the mild hypercholesterolemia as a qualification criterion. Thirty enrolled patients received simvastatin therapy (20 or 40 mg/day) for at least 1 year before the study (simvastatin “+” group). The remaining 30 patients, who did not receive any statin during the last year, were considered as a control group (simvastatin “−” group). All eligible women were in a good health, based on the physical examination and standard laboratory tests. The potential causes of secondary cholesterol level elevation (hypothyroidism, chronic steroids intake, liver and bile ducts disorders, nephrotic syndrome) were not noticed in the study group. Presence of any other diseases that were able to interfere with bone and joint metabolism especially immune disorders and obesity were considered as the exclusion criteria. All patients enrolled into the study did not use any drugs for osteoarthritis treatment and any other disease. The characteristic of the study group was given in Table 1. Dietary calcium and silicon intake was assessed by interview. The diet rich in these elements was an exclusion criterion from the study (diet rich in milky and dairy products, cereals, horsetail, rice, herbaceous plant) [15, 18].

**Table 1:** The characteristic of study group

	Simvastatin +	Simvastatin −	*t* test
Number	30	30	NA
Age (years)	60.7 ± 3.8	62.2 ± 3.9	*p* > 0.05
Mean total cholesterol level (mg/dl)	214.7 ± 12.7	211.8 ± 13.8	*p* > 0.05
Simvastatin 20/40 mg (*n*)	24/6	NA	NA

### Sample Collection

Venous blood was collected for the evaluation of calcium and silicon plasma level as well as simvastatin plasma level. Samples were obtained 3 h after oral simvastatin administration. All patients were fasted at least 3 h before blood sampling (from simvastatin administration to blood collection). Typically, simvastatin is administrated in the evening. Due to logistic reason, the simvastatin administration on the day of blood collection was moved to 4:00 p.m. The time of blood collection (3 h after drug administration) was established on the base of simvastatin mean plasma concentration-time curve which showed that the drug plasma level peaked at 3–4 h after administration [19]. After centrifugation, plasma samples were frozen at −70 °C and stored for further analysis.

Both total silicon and calcium concentrations in the plasma were determined spectrophotometrically using the method according to Wielkoszyński [20] and diagnostic kit (Cormay, Lublin, Poland), respectively. Wielkoszyński’s method was based on silicate reaction with molybdophosphoric acid. Tritrisol–Silicium (Merck) has been used as a standard, and standard curve was prepared ranging from 0 to 200 μmol/l, according to Wielkoszyński’s procedure.

Plasma concentration of simvastatin was determined using high-performance liquid chromatography (HPLC) with UV-Vis detector (Gilson, Inc., Middleton, USA) according to the Nagaraju and Vishnu’s method [21]. The mobile phase consisting of acetonitrile:phosphate buffer (pH = 4) in the ratio 75:25 *v*/*v* was delivered at the flow rate of 1.5 ml/min. Detection was performed at wavelength 238 nm. The analysis was carried isocratically on the Kromasil C18 column (4.6 × 250 mm, 5 μm). Plasma samples were purified using solid phase extraction (SPE) with Supel-Select HLB SPE Tubes (Sigma-Aldrich).

### Statistics

Student’s *t* test was applied for analysis of difference between calcium or silicon plasma level vs. study and control group (parametric distribution). Pearson correlation coefficient has been used to establish the relationship between simvastatin, silicon, and calcium plasma level of postmenopausal women. All hypotheses were verified at the significance level of *p* < 0.05. InStat GraphPad (La Jolla, CA, USA) was applied for statistical analysis.

## Results

**Fig. 1:**
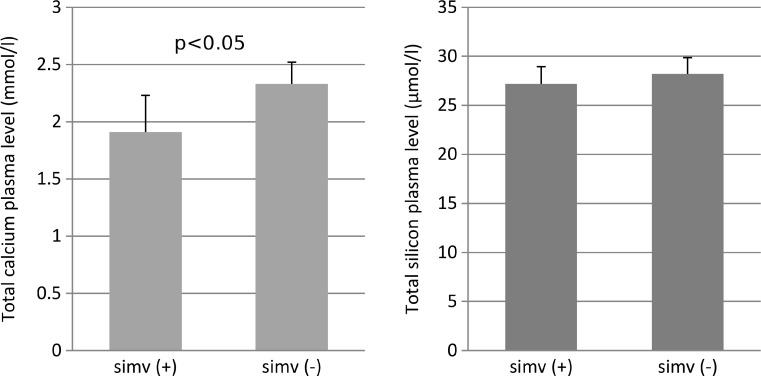
Total calcium and silicon plasma levels in postmenopausal women treated with simvastatin (simv +) in comparison with non-simvastatin group (simv −). The significant decrease of calcium level was noticed in simv + group. The difference between silicon plasma levels did not reach statistical significance. Student’s *t* test

Total plasma calcium level was significantly lower in female patients receiving simvastatin in comparison with non-statins group (*p* < 0.05; Fig. 1). Calcium concentration of simvastatin “+” group was below the normal range of applied method (normal range 2.1–2.6 mmol/l). The difference in silicon plasma level between analyzed study groups did not reach statistical significance (*p* > 0.05; Fig. 1). Plasma silicon concentration of both study groups remained within the normal range of the applied method (a reference range is 12.02–30.07 μmol/l). The mean of free simvastatin level in the plasma was 9.02 ± 1.18 ng/ml. The correlation analysis showed a significant positive relationship between silicon and simvastatin level (*r* = 0.3, *p* = 0.03). There was statistically insignificant (*r* = −0.2, *p* = 0.1) negative correlation between calcium and simvastatin plasma level.

## Discussion

The pathogenesis of osteoarthritis in postmenopausal women still remains unclear. Simvastatin, a lipid-lowering agent, is the most prescribed drug in the population of postmenopausal patients. The objective of our study was to evaluate the simvastatin effect on calcium and silicon plasma level in postmenopausal, osteoarthritic female patients.

Previous studies have shown that calcium and silicon are significant elements involved in bone and joint functioning, especially in older people [9, 12]. Calcium is strongly associated with bone and joint health. It was reported that women with low calcium intake more often suffered from osteoporosis and osteoarthritis [1]. Bergillos-Meca et al. [22] showed that the bioavailability of calcium was significantly higher in fermented milk containing the probiotic bacteria *Lactobacillusfermentum* D3. Our results suggest that simvastatin can contribute to the decrease of calcium concentration in the plasma. The negative correlation between simvastatin vs. calcium plasma levels was observed; however, it did not reach statistical significance. These findings are surprising in the light of previous studies that revealed lower incidence of fracture after statin use. The presence of low calcium in the blood usually is associated with the increased risk of vertebral fractures [1], but our results indicated the reduction of calcium in simvastatin-treated group. Tikiz et al. [23] reported that simvastatin treatment affects bone metabolism positively in a short time period. Clockaerts et al. [24] showed that statin use is strongly associated with more than 50 % reduction in progression of osteoarthritis of the knee. Observed decrease of calcium plasma level in simvastatin-treated group theoretically can be due to the intense use of calcium for bone recovery. Unfortunately, other study did find neither the stimulation of bone formation by simvastatin nor an elevation of osteoblast activity under in vivo conditions [25].

The dietary consumption of trace elements including silicon has been previously correlated with bone mass [12, 25]. Decreased incidences of bone fracture have been found in Asians and Indians, whose foods are relatively rich in various nutrients including silicon [26]. On the basis of the study performed on ovariectomized rats, Kim et al. [27] put forward the hypothesis that silicon supplementation can prevent osteoporosis in postmenopausal women whose calcium intake is insufficient. It has been found that oral silicon administration results in an increase in the bone density and improves bone formation [28]. Jugdaohsingh et al. [12] suggested that higher dietary silicon intake in men and young women might be beneficial in their skeletal health. They suggested as well that hormonal changes after menopause attenuate any positive effect on the bone. Silicon intake is strongly associated with bone mineral density (BMD) in premenopausal women, whereas no correlation has been found in postmenopausal women [28]. To the best of our knowledge, this is the first study evaluating the effect of simvastatin on silicon plasma level. Although no significant difference in silicon plasma level between simvastatin-treated and non-treated group was found, the positive correlation between simvastatin and silicon plasma levels can suggest that the statin is one of the potential factor affecting silicon concentration in the plasma. Nevertheless, the correlation between silicon plasma level and simvastatin is weak, probably due to the fact that the simvastatin contains silicon dioxide as an inactive ingredient. Due to unclear metabolism of silicon, the direct mechanism of statins on silicon plasma level is still the subject of speculation. Further in vivo and in vitro studies should be focused on explanation of molecular mechanism of simvastatin and other statins influence on calcium and silicon plasma levels.

## Abbreviations

BMD: Bone mineral density
BMP: Bone morphogenetic protein
HMG-CoA: 3-Hydroxy-3-methylglutaryl-coenzyme A reductase
HPLC: High-performance liquid chromatography
IL: Interleukin
MMP: Matrix metalloproteinase
SPE: Solid phase extraction

## References

[1] Alfred T, Ben-Shlomo Y, Cooper R, Hardy R, Cooper C, Deary IJ, Gunnell D (2013). Genetic markers of bone and joint health and physical capability in older adults: the HALCyon programme. Bone.

[2] Cunningham CC, Mills E, Mielke LA, O’Farrel LK, Lavelle E, Mori A, McCarthy GM (2012). Osteoarthritis-associated basic calcium phosphate crystals induce pro-inflammatory cytokines and damage-associated molecules via activation of Syk and P13 kinase. Clin Immunol.

[3] Zhang C-L, Dai L-Y, Jiang L-S, Qiu S (2012). Differences in subchondral cancellous bone between postmenopausal women with hip osteoarthritis and osteoporotic fracture. Arthritis Rheum.

[4] Nakashima M, Sakai T, Hiraiwa H, Hamada T, Omahi T, Ono I, Inukai N (2012). Role of S100A12 in the pathogenesis of osteoarthritis. Biochem Biophys Res Commun.

[5] Conaghan PG (2012). The effects of statins on osteoarthritis structural progression: another glimpse of the Holy Grail?. Ann Rheum Dis.

[6] Giorgi MA, Caroli C, Arazi HC, Di Girolamo G (2011). Pharmacogenomics and adverse drug reactions: the case of statins. Expert Opin Pharmacother.

[7] Garrett IR, Esparza J, Chen D, Zhao M, Gutierrez G, Escobado A, Horn D (2000). Statins mediate their effects on osteoblasts by inhibition of HMG-CoA reductase and ultimately BMP-2. J Bone Miner Res.

[8] Montagnani A, Gonnelli S, Cepollaro C, Pacini S, Compagna MS, Franci MB, Lucani B (2003). Effect of simvastatin treatment on bone mineral density and bone turnover in hypercholesterolemic postmenopausal women: a 1 year longitudinal study. Bone.

[9] Mundy GR, Guise TA (1999). Hormonal control of calcium homeostasis. Clin Chemistry.

[10] Kazantziz G (2004). Cadmium, osteoporosis and calcium metabolism. Biometals.

[11] Nazıroğlu M, Senol N, Ghazizadeh V, Yürüker V (2014). Neuroprotection induced by N-acetylcysteine and selenium against traumatic brain injury-induced apoptosis and calcium entry in hippocampus of rat. Cell Mol Neurobiol.

[12] Jugdaohsingh R, Tucker KL, Qiao N, Cupples LA, Kiel DP, Powell JJ (2004). Dietary silicon intake is positively associated with bone mineral density in men and premenopausal women of the Framingham offspring cohort. J Bone Miner Res.

[13] Zhang Y, Bradley AD, Wang D, Reinhardt RA (2014). Statins, bone metabolism and treatment of bone catabolic diseases. Pharmacol Res.

[14] Kang SC, Kim JH, Kim MH (2012). Effects of *Astragalus membranaceus* with supplemental calcium on bone mineral density and bone metabolism in calcium deficient ovariectomized rats. Biol Trace Elem.

[15] Jugdaohsingh R (2007). Silcon and bone health. J Nutr Health Aging.

[16] Jugdaohsingh R, Sripanyakorn S, Powell JJ (2013). Silicon absorption and excretion is independent of age and sex in adults. Br J Nutr 28.

[17] American College of Rheumatology Subcommittee on Osteoarthritis Guidelines. (2000). Recommendations for the medical management of osteoarthritis of the hip and knee: 2000 update. Arthritis Rheum.

[18] Kostecka M (2014). The role of healthy diet in the prevention of osteoporosis in perimenopausal period. Pak J Med Sci.

[19] Ding MJ, Yuan LH, Yun L, Wang S, Wu XL, Liu J, Ma KF (2011). Pharmacokinetics and bioequivalence study of simvastatin orally disintegrating tablets in Chinese healthy volunteers by LC-ESI-MS/MS. Bioequiv Availab.

[20] Wielkoszyński T (2000). Modified spectrophotometric method of silicon determination in biological material. Diagn Lab.

[21] Nagaraju P, Vishnu Z (2010). A validated reverse phase HPLC method for the simultaneous estimation of simvastatin and ezetimibe in pharmaceutical dosage forms. J Glob Pharma Technol.

[22] Bergillos-Meca T, Navarro-Alarcón M, Cabrera-Vique C, Artacho R (2013). The probiotic bacterial strain Lactobacillus fermentum D3 increases in vitro the bioavailability of Ca, P, and Zn in fermented goat milk. Biol Trace Elem Res.

[23] Tikiz C, Tikiz H, Tanlei F, Gumuser G, Tuzun C (2005). Effects of simvastatin on bone mineral density and remodelling parameters in postemenopausal osteopenic subject: 1-year follow-up study. Clin Rhematol.

[24] Clockaerts S, Osch GJVM, Bastiaansen-Jenniskens YM, Verhaar JAN, Glabbeek FV, Meurs JBV, Kerkhof HJM (2012). Statin use is associated with reduced incidence and progression of knee osteoarthritis in the Rotterdam study. Ann Rheum Dis.

[25] Von Stechow D, Fish S, Yahalom D, Bab I, Chorev M, Müller R, Alexander JM (2003). Does simvastatin stimulate bone formation in vivo?. BMC Musculoskelet Disord.

[26] Jugdaohsingh R, Anderson SH, Tucker KL, Elliott H, Kiel DP, Thompson RP, Powell JJ (2002). Dietary silicon intake and absorption. Am J Clin Nutr.

[27] Kim MH, Bae YJ, Choi MK, Chung YS (2009). Silicon supplementation improves the bone mineral density in calcium-deficient ovariectomized rats by reducing bone resorbtion. Biol Trace Elem Res.

[28] Macdonald HM, Hardcastle AC, Jugdaohsingh R, Fraser WD, Reid DM, Powell JJ (2012). Dietary silicon interacts with estrogen to influence bone health: evidence from the Aberdeen Prospective Osteoporosis Screening Study. Bone.

